# Updates on the Epidemiology of the Human T-Cell Leukemia Virus Type 1 Infection in the Countries of the Eastern Mediterranean Regional Office of the World Health Organization with Special Emphasis on the Situation in Iran

**DOI:** 10.3390/v14040664

**Published:** 2022-03-23

**Authors:** Mohammad Reza Hedayati-Moghaddam, Reza Jafarzadeh Esfehani, Hiba El Hajj, Ali Bazarbachi

**Affiliations:** 1 Blood Borne Infections Research Center, Academic Center for Education, Culture & Research (ACECR), Razavi Khorasan Branch, Mashhad 91775-1376, Iran; drhedayati@acecr.ac.ir (M.R.H.-M.); drrezajafarzadeh@yahoo.com (R.J.E.); 2 Department of Experimental Pathology, Microbiology and Immunology, Faculty of Medicine, American University of Beirut, Beirut 1107 2020, Lebanon; he21@aub.edu.lb; 3 Department of Internal Medicine, Faculty of Medicine, American University of Beirut, Beirut 1107 2020, Lebanon; 4 Department of Anatomy, Cell Biology and Physiological Sciences, Faculty of Medicine, American University of Beirut, Beirut 1107 2020, Lebanon

**Keywords:** human T-lymphotropic virus 1, Eastern Mediterranean Region, prevalence, epidemiology, Asian countries of EMRO, African countries of EMRO

## Abstract

Background: The epidemiology and prevalence of the Human T-cell leukemia virus type-1 (HTLV-1) infection represent a recommended priority by global health agencies. An in-depth revision to update the status of this infection in countries including those of the Eastern Mediterranean Regional Office (EMRO) of the World Health Organization is hence required. Methods: Ninety-seven studies evaluating the HTLV-1 infection in low- and high-risk populations in EMRO countries were retrieved from the international electronic databases and were used to assess the epidemiological status of the infection in these countries. Results: Most epidemiologic reports were published from Iran, with more than 50% of Iranian prisoners and around 4% of healthy individuals reported to have the infection. In Egypt, a considerable prevalence of the virus spans around 1.11% of blood donors. Foci of HTLV-1 infection are also present in some countries and require a careful epidemiological evaluation. In the other EMRO countries, a lower prevalence that does not exceed 1% was reported. Conclusion: The epidemiology and prevalence of HTLV-1 in the EMRO countries require a tight revision and update. Published studies reveal a scarce distribution of the virus in the African countries of EMRO, while a lower prevalence is denoted in the Asian countries of EMRO, except in Iran, where the prevalence is high.

## 1. Introduction

Human T-cell leukemia virus (HTLV) types 1 and 2 are the first identified human retroviruses. HTLV-1 was isolated from cultured lymphocytes of a patient with cutaneous lymphoma in 1979 [1], while HTLV-2 was found in a patient with hairy cell leukemia in 1982 [2]. HTLV-1 infects approximately 20 million people worldwide [3]. However, the endemic areas are clustered adjacent to regions where the infection is not common. The highest prevalence of HTLV-1 is reported in different continents; Asia, Japan, and Iran witnessed the highest prevalence of the virus. In Europe, Romania is highly endemic for the virus. The virus is prevalent in Intertropical West and South African countries, South America, and the Caribbean area [4]. In North America and Western Europe, HTLV-1 is predominant in migrants from endemic areas [5,6]. HTLV-1 infection was also reported in Melanesia, some islands in Oceania, and Central Australia [4,7,8].

HTLV-1 can be transmitted vertically from a mother to her child primarily by prolonged breastfeeding [9], through unprotected sexual intercourse mainly from male to female [10], by blood exposure via infected lymphoid cells [11], or by sharing needles between persons who use drugs [12]. HTLV-1 is the etiologic agent of a range of acute, chronic, and inflammatory disorders. Among these, adult T-cell leukemia-lymphoma (ATL) and the HTLV-1-associated myelopathy/tropical spastic paraparesis (HAM/TSP) are significant life-threatening morbidities [13]. In addition, it was demonstrated that the infection is associated with increased odds of developing diseases such as bronchitis, bronchiectasis, eczema, seborrheic dermatitis, and urinary tract infections which adversely affect the quality of life [14]. Moreover, the coinfection of HTLV-1 with other pathogens such as *Mycobacterium tuberculosis* and human immunodeficiency virus (HIV) was linked with a worse clinical course [15,16]. Likewise, pieces of evidence suggested that HTLV infection has an adverse effect on mortality even regardless of the effect of the increased number of inflammatory conditions [14]. To date, prophylactic measures are still lacking, and treatment strategies combatting this infection are not satisfactory. Moreover, the epidemiology of HTLV-1 remained obscure in certain areas and was underestimated or not updated in various parts of the world. Expanding the epidemiological studies to update the prevalence of HTLV-1 is one of the recent recommended actions by the HTLV-1 task force of the Global Virus Network [3]. The most recent World Health Organization technical guideline on HTLV-1 infection highlighted the same issue. The report emphasizes the need for epidemiological studies especially beyond the endemic areas, as migration contributed to increased detection of the infection [17]. The present review focused on the epidemiological studies on the distribution of HTLV-1 infection over the last two decades, based on the published reports on low-and high-risk populations in countries of the Eastern Mediterranean Regional Office (EMRO) of the World Health Organization.

## 2. Material and Methods

International electronic databases, including PubMed, Scopus, Web of Science, Index Medicus for the Eastern Mediterranean Region, and the Islamic World Science Citation Index, were searched until August 2021. The search strategy was as follows: (“human T-lymphotropic virus” OR “human T-cell lymphotropic virus”, OR “human T-cell leukemia-lymphoma virus” OR “HTLV”) AND (“Eastern Mediterranean” OR “Middle East” OR “North Africa” OR the names of each of the countries in the region) with no limitation to a specified field. Studies investigating HTLV-1 prevalence among populations at low and high risk of exposure to HTLV-1 were selected. Low-risk populations included healthy people, blood donors, pregnant women, and household-based survey participants. High-risk groups consisted of multi-transfused patients (such as hemodialysis, thalassemia, hemophilia, and malignancies), people who inject drugs (PWID), prisoners, and patients with HIV or sexually-transmitted diseases. The studies were selected based on HTLV-I detection using serological screening tests such as enzyme-linked immunosorbent assay (ELISA) or chemiluminescent immunoassay, with or without confirmation by complementary techniques such as immunoblotting or polymerase chain reaction (PCR).

The Eastern Mediterranean Region (EMR) comprises 15 Asian countries (Afghanistan, Bahrain, Iran, Iraq, Jordan, Kuwait, Lebanon, Oman, Pakistan, Palestine, Qatar, Saudi Arabia, Syria, the United Arab Emirates, and Yemen) and 7 African countries (Djibouti, Egypt, Libya, Morocco, Somalia, Sudan, and Tunisia) with a population of nearly 679 million individuals [18]. Studies published from January 2001 to August 2021 were included. Microsoft Excel (2010) was used to calculate the prevalence rates, and Epi Info 6.04b (CDC, USA) was used to compare the rates among males and females, using the chi-square test in each study. A *p*-value less than 0.05 was considered statistically significant.

## 3. Results and Discussion

**Figure 1:**
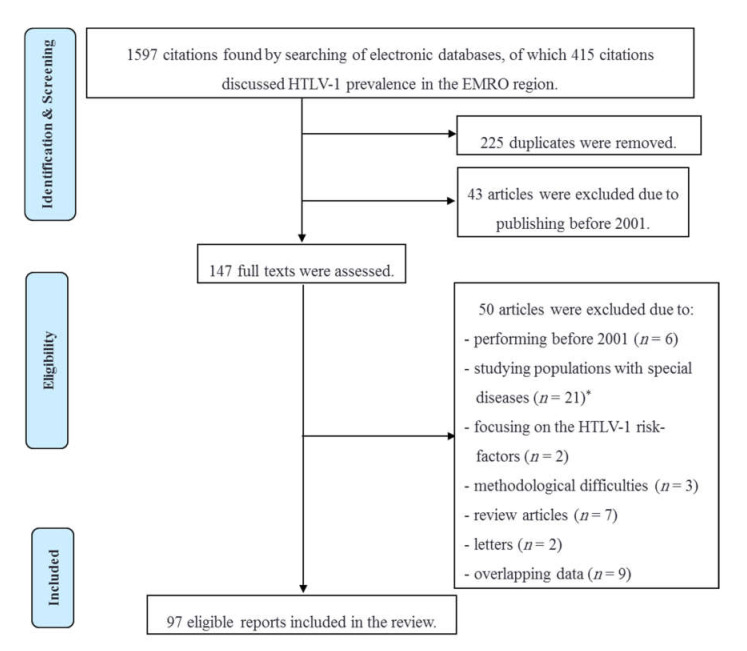
Study selection for the review of HTLV-1 infection epidemiology in the countries of the Eastern Mediterranean Regional Office of the World Health Organization. * Patients with dermatological, hematological, rheumatological, or autoimmune disorders; carcinomas; infectious diseases other then HIV/AIDS; chronic diseases such as cardiovascular disorders and diabetes Melitus, etc.

A total number of 1597 citations were identified in the five electronic databases. By screening the titles and abstracts, 190 non-duplicated citations discussing HTLV-1 prevalence among low- and high-risk populations in the EMRO region were identified. Forty-three articles published before 2001 were excluded, and the full texts of 147 remaining documents were reviewed. Forty-one papers were not included in this review. The reasons for excluding showed in Figure 1. Furthermore, nine surveys with the same studied cases, methods, and results were considered as overlapping and thus excluded. Finally, 97 non-overlapping original articles were included. They comprised 71 surveys from Iran; 15 from Saudi Arabia; 2 from Afghanistan, Lebanon, and Qatar; and 1 from Egypt, Iraq, Jordan, Kuwait, and Pakistan.

### 3.1. HTLV-1 Infection in Iran

#### 3.1.1. HTLV-1 Infection in Iranian Low-Risk Population

**Figure 2:**
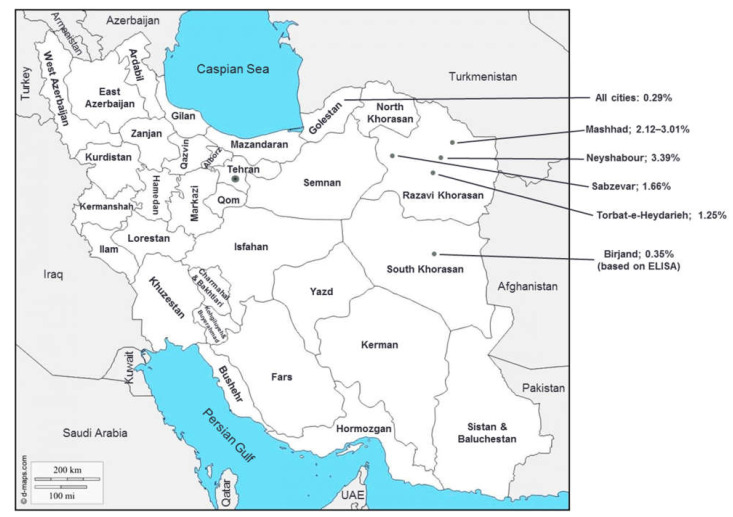
General population-based reports of HTLV-1 infection prevalence in Iran (References [19,20,21,22,23,24,30], source of background map: https://d-maps.com/carte.php?num_car=5494&lang=en, accessed on 12 February 2022).

The highest number of published reports on HTLV-1 infection in Southwest Asia was from Iran. However, general population-based reports of HTLV-1 infection were only identified from three among 31 provinces, including Razavi Khorasan, South Khorasan, and Golestan in the north and east parts of the country (Figure 2) [19,20,21,22,23]. Mashhad, the capital of Razavi Khorasan province, and its neighbor city, Neyshabour, in the northeast of Iran, were previously introduced as HTLV-1 endemic districts in Iran [24,25,26]. Later investigations confirmed that these two cities are still endemic regions for the infection. The overall HTLV-1 prevalence was estimated at 2.12% (35/1654, 95% CI: 1.48–2.93%) among the general population of Mashhad [21]. Likewise, the HTLV-1 prevalence reached 7.25% (35/483) of individuals referred to a referral laboratory in Neyshabour and 1.06% (2/189) in screens of subgroups including pregnant women or candidates for a surgical operation in that region [27]. Additionally, many cases with HAM/TSP and ATL were reported in this area [28,29]. Recent investigations revealed an HTLV-1 prevalence of 1.66% (24/1445) and 1.25% (5/400) in healthy populations of Sabzevar and Torbat-e-Heydarieh cities (located in Razavi Khorasan province), respectively [19,22]. Lower rates of HTLV-1 infection were reported among the general population of Birjand city, the capital of South Khorasan province (0.35%, 12/3441, based on ELISA results) and Golestan province (0.29%, 6/2034), respectively (Table 1) [20,23].

Recent phylogenetic and phylodynamic analysis of the long terminal repeat region of HTLV-1 in blood samples of 100 seropositive individuals from the north and south parts of Iran revealed that Iranian isolates belonged to the globally-distributed HTLV-1 Cosmopolitan subtype A [61]. These results concluded that most infections in Iran presumably occurred following the Mongol attack, before the 15th century, and were further facilitated by the ancient Silk Road linkage starting from China and continuing toward Turkey.

In order to check the published literature related to HTLV-1 infection prevalence among Iranian blood donors, the studies published until August 2011 were previously reviewed, and the prevalence of infection was estimated as low as 0.12% (95% CI: 0.05–0.29%); it ranged from 0.015% in the southwest to 0.38% in the northeast of Iran [62]. A meta-analysis by Azami et al. estimated the prevalence of HTLV-1 infection at 0.20% (95% CI: 0.15–0.26%) based on reviewing 34 studies comprising 3,626,364 blood donors published from 1996 to 2017 [63]. Regular screening of all donated blood units for antibodies against HTLV-1/2 followed by confirmatory tests was introduced in northeastern Iran in 1995 [64] and then extended to some provinces in the north and northwest parts of the country. These tests were performed among first-time and regular blood donors, and as a result, many surveys revealed a gradual decrease in HTLV-1 prevalence among Iranian blood donors from 0.13% in 2009 to 0.03% in 2018 (Table 1) [31,32,63]. A declining trend in the infection rate among blood donors was documented in Razavi Khorasan [41,42,45,47] as well as other provinces of Iran [48,51]. However, over time, no change was observed in the HTLV-1 infection rate among the general population of Razavi Khorasan province [21,26,30]. Statistically significant higher rates of HTLV-1 infection were reported in Iranian females as compared to males [30,31,32,41,42,44,45,46,51]. In that sense, a recent meta-analysis estimated a three-fold higher infection rate among female blood donors as compared to male donors (0.64%, 95% CI: 0.13–3.01% and 0.20%, 95% CI: 0.15–0.27%, respectively) [63]. However, some investigators could not find any differences between both sexes regarding HTLV-1 prevalence in Iran (Table 1); keeping the gender-related infection with this virus a controversial topic requires more epidemiological investigation. Considering a national screening program for mothers to avoid transmission of the virus through breastfeeding could be suggested as a preventive strategy. Furthermore, most of the surveys among the low-risk Iranian population revealed significantly greater rates of HTLV-1 infection among people with an age range between 40 and 60 years old, as compared to younger people in the bracket of 15 to 25 years old [19,20,21,22,24,27,31,32,42,44,45,51,56]. Our previous study among the general population of Mashhad showed an 18-fold higher infection rate among individuals older than 55 years (9.14%, 17/186), compared to people younger than 15 years (0.52%, 2/387). The logistic regression analysis revealed that the participants’ age could significantly affect the risk of infection (Odds ratio = 4.3, 95% CI: 1.5–12.3) [21]. Similarly, Emadi et al. reported an HTLV-1 prevalence in 4 per 100,000 among 263,465 blood donors aged between 18 and 25 years old, while 66 per 100,000 donors of 46 to 65 years old [51]. Conversely, some studies with considerable sample sizes (from 407 to 35,067 individuals) reported no significant relationship between participants’ age and the infection rate [23,36,47,54]. An age- and sex-matched case-control study to assess the HTLV-1 risk factors among “first-time” Iranian blood donors was performed during the period 2011–2012 [65]. The regression analysis revealed that the city of birth, low income, low educational level, history of blood transfusion, and drug abuse were correlated with HTLV-1 infection. In addition, living in the northeastern of Iran [31], being married [21,31,42,48], populated family [27], history of blood transfusion [21,27], hospitalization [21,27], surgery [19,21], wet cupping [21], and imprisonment [19] seemingly correlate with HTLV-1 infection among Iranian population. Moreover, many studies showed that “first-time” blood donors are more likely to be HTLV-1 seropositive than regular donors [31,32,42,47,48,51], which could be because of those who are positive for the infection are not allowed to donate again.

In terms of clinical findings among HTLV-1 infected individuals in Iran, most studies focused on the neurologic manifestations of the disease. Shoeibi et al. reported that gait disturbances followed by sensory and urinary complaints are the most common manifestation in HAM/TSP patients in Mashhad, Iran. In contrast, other clinical symptoms, including back pain, constipation, and erectile dysfunction, are not commonly reported. The hypertonicity is mainly reported in lower limbs, and most of the patients develop brisk tendon reflexes in all limbs. In terms of sensory findings, foot paresthesia was a common compliment, and impairment of vibration sensation was more prominent in lower limbs [29]. While cranial nerves and cerebellum involvement were reported in HTLV-1 infected patients, this finding was not reported in Iranian HAM/TSP patients [29]. Among Iranian ATLL patients, laboratory findings including leukocytosis, neutropenia, and lymphocytosis were common findings. Moreover, elevated serum alkaline phosphatase and lactate dehydrogenase were reported in 80% and 75% of these patients in Mashhad city [28].

#### 3.1.2. HTLV-1 Infection among High-Risk Iranian Population

HTLV-1 prevalence was also investigated among high-risk Iranian populations, including patients with thalassemia or hemophilia, those undergoing hemodialysis, HIV-positive individuals, PWID, and prisoners. Significant high rates of HTLV-1 infection (3.24%, 95% CI: 2.19–4.78%) were previously estimated among Iranian multi-transfused patients [66]. Currently, the rate of HTLV-1 prevalence varies between 0.18% and 11.28% among thalassemia patients and 0 to 14.49% among the hemodialysis group (Table 2). The highest rates among hemodialysis patients were reported from Razavi Khorasan (5.93%, 8/135 and 14.49%, 20/138) and Charmahal and Bakhtiari provinces (6.54%, 7/107) [67,68,69]. Besides, the highest rates among thalassemia patients were reported from Tehran (6.29%, 11/175 and 11.28%, 29/257) and Charmahal and Bakhtiari provinces (6.80%, 17/250) [49,69,70,71]. Regarding the considerable rate of HTLV-1 infection among patients receiving blood products, more restrictive preventive measures such as screening and leukoreduction programs in blood banks should be considered to reduce the infection transmission through the infected products. Furthermore, considerably high rates of infection were documented in persons experiencing drug dependence and/or HIV-positive individuals in Razavi Khorasan (51.49%, 52/101 and 15%, 3/20, respectively) [12,72], and Khuzestan provinces (16.35%, 17/104) [73]. Rowhani-Rahbar et al. reported a very high rate of HTLV-1 infection (51.49%) among 101 prisoners with a dependence on a substance in the central prison of Mashhad in 2001, especially among subjects who shared their syringes (Odds ratio = 2.5, 95% CI: 1.1–5.7) [12]. Moreover, Khajedaluee et al. reported that HTLV-1 infection occurred in 2% of 1114 inmates of two central prisons in Mashhad in 2008 [74]. Nearly two-fifths of the responders were drug users, and the infection was associated with a history of drug dependence [74]. Reducing HTLV-1 transmission could be improved by avoiding sharing needles or syringes and using sterile devices provided by healthcare systems. Moreover, considering the HTLV-1 infection as a sexually transmitted disease, condom use should be encouraged to prevent sexual transmission from the infected patients, especially those who engage in risky sexual relationships [75].

### 3.2. HTLV-2 Infection in Iran

Screening 1654 serum samples from the general population of Mashhad did not demonstrate any HTLV-2 positive cases [98]. This was asserted by the results of nested PCR using specific external and internal primers to detect HTLV-2 provirus in 50 Iranian HTLV-1-positive blood donors, who showed sero-indeterminate results in the Western blotting test. Ten cases with positive PCR results for HTLV-1 were identified, while all samples were negative for HTLV-2 [99]. On the other hand, there is no evidence regarding HTLV-2 infection incidence among the high-risk Iranian population. However, the prevalence of this infection was considerably high among PWID in western countries [100].

### 3.3. HTLV-1 Infection in EMRO Countries except for Iran

#### 3.3.1. HTLV-1 Infection in Asian Countries of EMRO

In countries of Southwest Asia other than Iran, HTLV-1 infection seems non-endemic (Table 3). In a survey among the general population of five provinces in Afghanistan, anti-HTLV-1 antibodies were detected in 0.64% of 466 participants using ELISA. However, no complimentary test was further performed to confirm these results [101]. The highest infection rate (1.59%, 2/126) was observed among people older than 45 years, but the rate was associated with neither the participants’ gender nor the geographical region. Another survey conducted in four cities of Afghanistan showed no evidence of HTLV-1 seropositivity in 80 hemophilia screened patients [102].

In a cross-sectional survey performed in Qatar, 0.18% of nearly 200,000 blood samples donated from 2013 to 2017 showed HTLV-1/2 seroreactivity in enzyme-linked and line immunoassays, and the trend for this infection was increasing from 0.08% to 0.23% [110]. Likewise, Ibrahim et al. demonstrated a rate of 0.26% (39/15239) HTLV-1/2 seropositivity among samples collected in Iraqi blood banks using enzyme-linked and chemiluminescent immunoassays [104]. No or meager seroprevalence rates of HTLV-1 infection were reported in blood donors from Jordan [105], Kuwait [106], Lebanon [107,108], Pakistan [111], and different parts of Saudi Arabia [109,114,115,116,117,118,120,122,124,125]. In Lebanon, no HTLV-1 positive cases were found initially among blood donors and even in high-risk groups such as PWID, hemophilia patients, or those with malignancy who received multiply blood transfusions [107]. Bitar et al. later reported two cases of acute ATL diagnosed in Lebanon, one from the Lebanese origin and the second from the Romanian origin. In both patients, Western blotting confirmed HTLV-1 seropositivity, and the HTLV-I oncoprotein Tax expression was documented in the leukemic cells [127]. Upon screening of Lebanese patient family members, seven direct family members were HTLV-I positive, and four were regular blood donors [127].

HTLV-1 infection rates were associated with the nationality of participants in most Arab countries. In Saudi Arabia, slightly higher infection rates were reported among non-Saudi blood donors (0.074–0.113%) as compared to Saudi donors (0.046–0.055%) [112,113]. Nevertheless, other studies showed a decreasing trend in HTLV-1 prevalence among blood donors of Saudi Arabia [113,116]. Similarly, the infection rate among 81,699 non-Qatari (including Arab and non-Arab) blood donation volunteers was 0.03%, but only one positive case was identified among 42,567 Qatari national donors (0.002%) [109]. Conversely, one of 8561 Kuwaiti blood donors was HTLV seropositive, and no positive HTLV cases were detected among 4237 non-Kuwaiti Arab donors [106].

**Figure 3:**
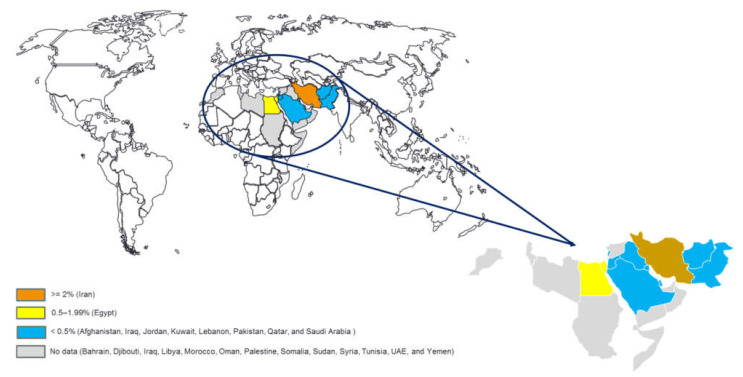
Map of the geographical distribution of HTLV-1 infection in countries of the Eastern Mediterranean Regional Office of the World Health Organization (drawn based on the epidemiological reports in the general population or blood donors included in this review).

Not a single report documented the prevalence of HTLV-1 infection in other Asian countries, including Bahrain, Oman, Palestine, Syria, United Arab Emirates, and Yemen of EMRO (Figure 3). However, sporadic cases were reported in Arab populations residing in other countries. Fawzi et al. reported one person with HTLV-1 among 1929 Syrian blood donors who resided in Qatar but did not find any seropositive cases among donors who originated from Jordan, Palestine, and Yemen [109]. Similarly, Bazarbachi et al. diagnosed four HTLV-1 positive ATL patients among Iraqi patients who sought medical care in Lebanon (manuscript in preparation). Although no formal epidemiological study was conducted in Iraq, epidemiological foci of HTLV-1 infection seemingly exist. Therefore, a tight screening to evaluate the incidence, prevalence, and burden of this viral infection is required in the Iraqi population.

As with Iran, the limited genotype data indicated that the most frequent subtype of HTLV-1 is the “transcontinental” subgroup of cosmopolitan (A) subtype over the EMRO region [4].

#### 3.3.2. HTLV-1 Infection in African Countries of EMRO

Only one epidemiologic survey in Egypt was conducted and reported one HTLV-1-positive case among 90 Egyptian blood donors during 2005–2006 (Table 3). Unfortunately, not a single epidemiological study from Djibouti, Libya, Morocco, Somalia, Sudan, and Tunisia was identified over the last two decades, leaving a gap in our knowledge on the status of HTLV-1 infection in these EMRO countries (Figure 3). However, it is noteworthy to mention that there are some reports regarding the infected individuals who originated from Somalia [109], Sudan [109], and morocco [128], which highlight the need for more epidemiological studies in this part of the EMRO countries.

### 3.4. Study Limitations

In the present study, we skimmed the known literature to evaluate the epidemiological status of HTLV-1 infection in the countries of the EMRO region. We relied on the scientific reports indexed in specific electronic databases. We also tried to include common international and regional databanks. Therefore, some reports about the prevalence of HLTV-1 infection may be lacking. These include indexed reports in other sources or data not included in any database but presented in conference proceedings, dissertations, organizational reports, and magazines. Reports considering HAM or ATL published from the EMRO region were also excluded.

Moreover, the heterogeneous studied population of the surveys included in the present review may make the comparison of the results difficult. In addition, confirmatory laboratory tests were not performed in some studies or were not the same across all the studies. The present review included the studies with even small sample sizes to cover the entire EMRO population as possible.

## 4. Conclusions

The epidemiologic data on HTLV-1 infection in most parts of the Eastern Mediterranean region are limited or lacking. The available published literature reveals that the prevalence of HTLV-1 infection in part of the Eastern Mediterranean region, except Iran, is low. The evidence on HTLV-1 prevalence in the African part of EMRO is scarce, and the epidemiological data in endemic regions are not updated. A more systematic and more frequent analysis of the status of HTLV-1 infection in EMRO countries is required, especially in countries such as Iraq and Morocco, where the HTLV-1 infection was identified and reflected the presence of foci in these countries. Finally, the longstanding infection control strategies, including the HTLV-1 screening of blood donors in most endemic regions, requires future validation by complementary techniques, such as PCR, to increase the specificity of the infection diagnosis.

## Figures and Tables

**Table 1:** Prevalence of HTLV-1 infection among low-risk population of Iran; 2001–2021.

Population	Province (City, Direction in Country)	Study Year	Sample Size	Participants’ Age; Ranges (Mean ± SD)	Male to Female Ratio	Lab Techniques	Total Prevalence (%)	Prevalence by Sex (%)			Ref.
								Males	Females	*p*-Value	
General population	Golestan (North)	2007	2034	(38.7 ± 16.50)	0.72	ELISA, WB	0.29	0.35	0.25	0.49	[20]
	Razavi Khorasan (Mashhad, Northeast)	?	9274	?	0.67	ELISA, PCR	3.01	2.34	3.46	0.002	[30]
		2009	1654	1–90	0.83	ELISA, WB, PCR	2.12	1.46	2.66	0.093	[21]
	Razavi Khorasan (Neyshabour, Northeast)	2002	1003	10–80 (32 ± ?)	0.68	ELISA, WB	3.39	2.96	3.68	0.539	[24]
	Razavi Khorasan (Sabzevar, Northeast)	2008	1445	5–88 (36.6 ± 15.6)	0.46	ELISA, PCR	1.66	2.42	1.31	0.128	[19]
	Razavi Khorasan (Torbat-e-Heydarieh, Northeast)	2011	400	14–89 (45.0 ± 16.8)	1.05	ELISA, PCR	1.25	1.46	1.03	0.09	[22]
	South Khorasan (East)	2013–2014	3441	14–70 (38.7 ± 14.50)	0.88	ELISA	0.35	0.25	0.44	0.34	[23]
Blood donors	Seven provinces	2009–2013	1,864,489	18–65 (34.8 ± 10.4)	10.32	ELISA, WB	0.10	0.09	0.18	<0.001	[31]
	Razavi Khorasan (Northeast)		628,667	?	?	ELISA, WB	0.21	?	?	?	
	West Azerbaijan (Northwest)		307,422	?	?	ELISA, WB	0.07	?	?	?	
	North Khorasan (Northeast)		79,035	?	?	ELISA, WB	0.06	?	?	?	
	Alborz (Center)		264,340	?	?	ELISA, WB	0.06	?	?	?	
	South Khorasan (East)		72,185	?	?	ELISA, WB	0.03	?	?	?	
	Gilan (North)		373,227	?	?	ELISA, WB	0.02	?	?	?	
	Ardabil (Northwest)		139,613	?	?	ELISA, WB	0.01	?	?	?	
	Seven provinces (East, North, Northeast, Northwest, Center)^1^	2010–2018	3,622,860	18–65	18.48	ELISA, WB	0.07	0.06	0.23	<0.001	[32]
	Bushehr (Southwest)	2002–2003	22,740	?	3.79	ELISA, WB	0.01	0.02	0	-	[33]
	Charmahal and Bakhtiari (Southwest)	2005–2006	800	?	?	ELISA, WB	0.50	?	?	?	[34]
	Fars (Shiraz)	?	500	?	15.97	ELISA	0.20	?	?	?	[35]
	Golestan (North)	2017	4226	males: (37.6 ± 7.9), females: (38.3 ± 8.9)		ELISA, WB, PCR	0.09	?	?	?	[36]
	Hormozgan (South)	2007–2008	1100	?	?	ELISA, WB, PCR	0.18	?	?	?	[37]
	Kermanshah (West)	2015	470	10–59	9.0	ELISA, WB	0	0	0	-	[38]
		2011	1000	(43.1 ± 19.8)	0.71	ELISA, WB	0.50	0	0.86	0.079	[39]
	Mazandaran (Babol, North)	2015–2016	503	19–61, males: (40.8 ± 9.8), females: (41.2 ± 11.4)	24.15	ELISA, PCR	0.20	0.21	0	-	[40]
	Razavi Khorasan (Mashhad, Northeast)	2001–2002	60,892	?	?	ELISA, WB	0.66	?	?	?	[12]
		2004–2006	232,648	18–65 (30 ± ?)	9.88	ELISA, WB	0.45	0.42	0.76	<0.001	[41]
		2006–2008	250,582	17–63 (38.3 ± 10.8)	11.74	ELISA, WB	0.40	0.36	0.88	<0.001	[42]
		2008–2009	79,687	17–65	?	ELISA, WB	0.38	?	?	?	[43]
		2009–2010	165,860	17–59 (39.9 ± 10.5)	13.71	ELISA, WB	0.26	0.23	0.70	<0.001	[44]
		2002–2013	983,000	(35 ± 10 to 41 ± 11)	14.63	ELISA, WB	0.30	0.28	0.48	<0.001	[45]
		2011–2013	174,662	?	12.90	ELISA, WB	0.19	0.16	0.56	<0.001	[46]
	Razavi Khorasan (Sabzevar, Northeast)	2009–2012	35,067	17–59 (38.1 ± 11.8)	9.06	ELISA, WB	0.14	0.13	0.26	0.095	[47]
	South Khorasan (East)	2005–2015	165,267	males: (29.5 ± ?), females: (32.8 ± ?)	11.55	ELISA, WB	0.03	0.03	0.02	0.038	[48]
	Tehran (North)	?	2000	?	?	ELISA, PCR	0.05	?	?	?	[49]
	West Azerbaijan (Northwest)	2005	2046	(31.9 ± 10.2)	14.04	ELISA, WB	0.34	0.26	1.47	0.074	[50]
		2009–2019	682,171	18–65	30.13	ELISA, WB	0.05	0.05	0.14	<0.001	[51]
Corneal donors	Nationwide	2005–2007	5533	2–84	4.19	ELISA, WB	0.67	0.58	1.03	0.105	[52]
Tissue donors	Nationwide	2002–2007	1548	0–66 (29.1 ± 11.6)	3.11	ELISA	1.61	1.45	2.12	0.369	[53]
Pregnant women	Razavi Khorasan (Mashhad, Northeast)	2010–2011	407	(26 ± ?)	?	ELISA, PCR	1.47	?	?	?	[54]
People who referred to a medical lab	Razavi Khorasan (Neyshabour, Northeast)	2009	483	5–84 (37.4 ± 15.4)	0.26	ELISA, WB	7.25	10.31	6.49	0.196	[27]
		2010–2014	8054	males: (46 ± 3), females: (51 ± 3)	0.24	ELISA	6.56	8.31	6.13	0.002	[55]
		2013–2014	1169 ^2^	2–35, (26.3 ± 5.4)	0.14	ELISA	3.34	6.38	2.92	0.058	[56]
		2011–2015	5724 ^3^	15–40 (28.2 ± 6.0)	?	ELISA	2.04	-	2.04	-	[57]
People who admitted to a referral hospital	Mazandaran (Sari, North)	2009–2010	1200	1–76	0.55	ELISA, WB	0.08	0.24	0	-	[58]
	Razavi Khorasan (Northeast)	2016–2017	758 ^4^	0–14 (6.4 ± 1.8)	1.14	ELISA, PCR	1.45	?	?	?	[59]
	Tehran (North)	2009–2011	219	13–84 (39.9 ± 16.5)	0.72	ELISA	1.83	1.09	2.36	0.641	[60]

(?) means not reported; (-) means not-applicable; ELISA: Enzyme-linked immunosorbent assay; WB: Western blotting; PCR; polymerase chain reaction. ^1^ Included Alborz, Ardabil, Gilan, North, South, and Razavi Khorasan, and West Azerbaijan. ^2^ Children and juveniles (2–35 years old), ^3^ Young females (15–40 years old), ^4^ Children (less than 15 years old).

**Table 2:** Prevalence of HTLV-1 infection among high-risk populations of Iran; 2001–2021.

Population	Province (City, Direction in Country)	Study Year	Sample Size	Participants’ Age; Ranges (Mean ± SD)	Male to Female Ratio	Lab Techniques	Prevalence (%)	Ref.
Hemodialysis patients	Busher (Southwest)	2003	101	?	?	ELISA, WB	0	[76]
	Charmahal and Bakhtiari (Southwest)	2005	107	18–90, (65 ± ?)	?	ELISA, WB	6.54	[69]
	Hormozgan (South)	2007–2008	40	?	?	ELISA, WB	0	[37]
	Kurdistan (Sanandaj, West)	2010	65	(45.1 ± ?)	?	ELISA, WB	0	[77]
	Mazandaran (Sari and Ghaemshahr, North)	2011	160	(59.1 ± 14.7)	1.0	ELISA, WB	0.63	[78]
	Razavi Khorasan (Mashhad, Northeast)	2009–2010	135	males: (43.5 ± 12.5), females: (50.5 ± 13.2)	0.99	ELISA, PCR	5.93	[67]
	Razavi Khorasan (Neyshabour, Northeast)	2012	138	12–84, (53.3 ± 17.9)	1.23	ELISA, WB	14.49	[68]
	South Khorasan (Birjand, East)	2010	41	(54.9 ± 16.5)	2.15	ELISA, WB	2.44	[79]
	Tehran (North)	?	150	24–88, (63.6 ± 13.4)	1.34	ELISA, WB	0.67	[80]
		2016–2017	174	17–86, (56 ± ?)	1.32	ELISA, PCR	1.15	[81]
	West Azerbaijan (Urmia, Northwest)	2006	95	(31.9 ± 10.2)	?	ELISA, WB	1.05	[50]
Thalassemia patients	Busher (Southwest)	2003	455	?	?	ELISA, WB	3.08	[76]
	Charmahal and Bakhtiari (Southwest)	2005	250	1–45, (25 ± ?)	?	ELISA, WB	6.80	[69]
	Fars (Shiraz, South)	?	200	?	?	ELISA	3.00	[35]
	Golestan (Gorgan, North)	2004–2005	181	1–25, (14.1 ± 6.5)	1.06	ELISA, WB	4.42	[82]
	Hormozgan (South)	2007–2008	163	?	?	ELISA, WB, PCR	3.07	[37]
	Isfahan (Center)	2007	150	1–49, (17.7 ± ?)	1.42	ELISA, WB	3.33	[83]
		2012	67	?	?	ELISA, PCR	1.49	[84]
	Kermanshah (West)	2011	116	(16.8 ± 6.6)	1.23	ELISA, WB	3.45	[39]
	Kurdistan (Sanandaj, West)	2010	46	(13.3 ± ?)	?	ELISA, WB	2.17	[77]
	Mazandaran (North)	2009	288	(21.5 ± 6.6)	0.91	ELISA, PCR	1.39	[85]
	Mazandaran (Tonekabon, North)	2015	80	?	?	ELISA, PCR	2.50	[86]
	Razavi Khorasan (Mashhad, Northeast)	2007	360	1–52, (11.6 ± 0.5)	1.38	ELISA	6.11	[87]
		2006–2013	100	5–46, (22.7 ± ?)	1.38	ELISA, PCR	4.00	[88]
	Tehran (North)	2003	175	(18.1 ± 1.0)	1.36	ELISA, WB	6.29	[70]
		2008–2010	257	?	0.89	ELISA, WB	11.28	[71]
		?	100	?	?	ELISA, PCR	8.00	[49]
Hemophilia patients	Busher (Southwest)	2003	86	?	?	ELISA, WB	0	[76]
	South Khorasan (East)	?	80	(21.3 ± 12.1)	25.67	ELISA, WB	1.25	[89]
		2010–2012	108	14–85, (27.7 ± 16.4)	14.43	ELISA	2.78	[90]
	West Azerbaijan (Northwest)	?	50	(10.3 ± ?)	6.14	ELISA, WB	0	[91]
Patients with combined factor 5 and 8 deficiency	Razavi Khorasan (Mashhad, Northeast)	2007	24	6–61, (26.9 ± 15.1)	2.0	ELISA, WB	0	[92]
HIV-positive patients	Isfahan (Center)	2010–2011	56	(37.0 ± 8.7)	5.22	ELISA	1.79	[93]
	Khuzestan (Ahwaz, Southwest)	2001–2003	104 ^1^	?	*	ELISA, WB	16.35	[73]
	Kurdistan (Sanandaj, West)	2010	130 ^1^	(27.2 ± ?)	239.0	ELISA, WB	0.77	[77]
	Razavi Khorasan (Mashhad, Northeast)	?	20	22–50, (36.4 ± 8.6)	4.0	ELISA, WB	15.0	[72]
	Tehran (North)	?	100	?	?	ELISA, PCR	5.00	[49]
People who inject drugs	Isfahan (Center)	2007–2008	150 ^2^	(30.7 ± 7.1)	74.0	ELISA	2.67	[94]
	Khuzestan (Ahwaz, Southwest)	2001–2003	104 ^1^	?	*	ELISA, WB	16.35	[73]
	Kurdistan (Sanandaj, West)	2010	130 ^1^	(27.2 ± ?)	239.0	ELISA, WB	0.77	[77]
		2010	110 ^3^	?	?	ELISA, WB	0.91	[77]
	Razavi Khorasan (Mashhad, Northeast)	2001	101 ^4^	(32.8 ± 8.9)	?	ELISA, WB	51.49	[12]
		2007–2008	62 ^5^	(34.3 ± ?)	30.0	ELISA	8.06	[95]
	Tehran (North)	?	180	9–67, (36.9 ± 9.2)	?	ELISA, WB	0	[96]
Prisoners	Razavi Khorasan (Northeast)	2001	101 ^4^	(32.8 ± 8.9)	?	ELISA, WB	51.49	[12]
		2008	1114 ^6^	males: (34.4 ± 10.9), females: (40.7 ± 14.2)	8.13	ELISA, PCR	1.97	[74]
	South Khorasan (Birjand, East)	2014–2015	300 ^7^	20–78, (37.4 ± 9.4)	*	ELISA, WB	0	[97]

(?) means not reported; * All cases were male; ELISA: Enzyme-linked immunosorbent assay; WB: Western blotting; PCR; polymerase chain reaction. ^1^ HIV-positive people who use drugs. ^2^ Including outpatients and hospitalized people who use drugs. ^3^ HIV-negative people who use drugs. ^4^ Imprisoned people who use drugs. ^5^ Hospitalized people who use drugs. ^6^ Including people who use drugs and others. ^7^ Including people who use and/or inject drugs.

**Table 3:** Prevalence of HTLV-1 infection low- and high-risk populations of countries in the Eastern Mediterranean Regional Office of WHO (except Iran); 2001–2021.

Population	Country (Province, City)	Study Year	Sample Size	Participants’ Age; Ranges (Mean ± SD)	Male to Female Ratio	Lab Techniques	Prevalence (%)	Ref.
General population	Afghanistan (5 provinces) ^1^	2015	466	25–70, (38.9 ± 12.0)	0.98	CMIA	0.64	[101]
Blood donors	Egypt (Cairo)	2005–2006	90	18–47, (29.3 ± 6.5)	6.50	ELISA, PCR	1.11	[103]
	Iraq (7 provinces) ^2^	2015	15,239	20–57, (36 ± ?)	?	ELISA, CMIA	0.26	[104]
	Jordan (Amman)	2009–2013	62,933	18–60	?	ELISA	0	[105]
	Kuwait (Nationwide)	2002	12,798	males: 20–56, (35 ± ?), females: 24–50, (30 ± ?)	?	ELISA, CMIA	0.01	[106]
	Lebanon (Beirut)	2000–2002	500	(38.7 ± ?)	3.17	ELISA	0	[107]
	Lebanon (Nationwide)	2001–2002	3529	(30.0 ± 8.9)	22.80	ELISA, WB, PCR	0.06 (WB), 0.03 (PCR)	[108]
	Qatar (Nationwide)	1991–2003	124,266	?	?	ELISA, WB	0.0002	[109]
	Qatar (Doha)	2013–2017	190,509	?	10.03	CMIA, LIA	0.18	[110]
	Pakistan (Rawalpindi)	2013	2100	18–60, (29.0 ± 9.3)	62.64	CMIA, LIA, PCR	0.19	[111]
	Saudi Arabia (Al-Khobar)	1995–2001	23,493	(33.8 ± ?)	?	ELISA, WB	0.05	[112]
	Saudi Arabia (Dammam)	1998–2001	13,443	?	?	ELISA, WB	0.06	[113]
	Saudi Arabia (Riyadh)	1999–2001	24,654	?	?	ELISA, WB	0.004	[114]
	Saudi Arabia (Riyadh)	2000–2002	24,173	(33.8 ± ?)	108.38	ELISA, LIA	0	[115]
	Saudi Arabia (Al-Hasa)	1997–2003	47,426	18–55	?	ELISA, WB	0.01	[116]
	Saudi Arabia (Jeddah)	?	30,000	?	?	ELISA, WB	0	[117]
		2006–2015	107,419	?	?	CMIA, WB	0	[118]
	Saudi Arabia (Al-Baha)	2009–2011	2807	16–66	*	ELISA	0.04	[119]
	Saudi Arabia (Aseer)	2012	4432	18–60, (30 ± ?)	*	ELISA	0	[120]
	Saudi Arabia (Aseer)	2012–2013	7267	18–60	278.50	ELISA	0	[121]
	Saudi Arabia (Qassim/Unaizah)	2013–2016	9460	18–48, (31.4 ± 8.7)	26.82	ELISA, PCR	0.10	[122]
	Saudi Arabia (Hail)	2016	361	<20 to >50	?	ELISA	2.22	[123]
	Saudi Arabia (Majmaah)	2015–2017	3028	18–61	44.6	ELISA	0.20	[124]
	Saudi Arabia (Buraidah)	2017–2018	4590	16–65	4.88	ELISA	0	[125]
	Saudi Arabia (Najran)	?	953	?	237.25	ELISA	0	[126]
Hemophilia patients	Afghanistan (four cities) ^3^	2017	80	2–38, (13.7 ± 9.8)	?	ELISA	0	[102]
	Lebanon (Beirut)	2000–2002	30	(37.4 ± ?)	*	ELISA	0	[107]
Malignancy patients with multiply transfusions	Lebanon (Beirut)	2000–2002	65	(30.5 ± ?)	1.32	ELISA	0	[107]
People who inject drugs	Lebanon (Beirut)	2000–2002	40	(50.3 ± ?)	3.0	ELISA	0	[107]

(?) means not reported; * All cases were male; CMIA: Chemiluminescent microparticle immunoassay; ELISA: Enzyme-linked immunosorbent assay; LIA: Line Immunoassay; WB: Western blotting; PCR; polymerase chain reaction. ^1^ Including Nangarhar, Herat, Mazar-e Sharif, Kandahar, and Kabul. ^2^ Including Baghdad, Karbala, Al-Qadisiyyah, Al-Najaf, Al-Muthanna, Al-Basrah, and Wasit. ^3^ Including Kabul, Herat, Mazar-i-Sharif, and Jalal Abad.
